# Prediction of incomplete immunization among under-five children in East Africa from recent demographic and health surveys: a machine learning approach

**DOI:** 10.1038/s41598-024-62641-8

**Published:** 2024-05-21

**Authors:** Zinabu Bekele Tadese, Araya Mesfin Nigatu, Tirualem Zeleke Yehuala, Yakub Sebastian

**Affiliations:** 1 Department of Health Informatics, School of Public Health, College of Medicine and Health Science, Samara University, Samara, Ethiopia; 2 Department of Health Informatics, Institute of Public Health, College of Medicine and Health Science, University of Gondar, Gondar, Ethiopia; 3 Department of Information Technology, Faculty of Science and Technology, Charles Darwin University, Darwin, Australia

**Keywords:** Vaccines, Paediatric research

## Abstract

The World Health Organization as part of the goal of universal vaccination coverage by 2030 for all individuals. The global under-five mortality rate declined from 59% in 1990 to 38% in 2019, due to high immunization coverage. Despite the significant improvements in immunization coverage, about 20 million children were either unvaccinated or had incomplete immunization, making them more susceptible to mortality and morbidity. This study aimed to identify predictors of incomplete vaccination among children under-5 years in East Africa. An analysis of secondary data from six east African countries using Demographic and Health Survey dataset from 2016 to the recent 2021 was performed. A total weighted sample of 27,806 children aged (12–35) months was included in this study. Data were extracted using STATA version 17 statistical software and imported to a Jupyter notebook for further analysis. A supervised machine learning algorithm was implemented using different classification models. All analysis and calculations were performed using Python 3 programming language in Jupyter Notebook using imblearn, sklearn, XGBoost, and shap packages. XGBoost classifier demonstrated the best performance with accuracy (79.01%), recall (89.88%), F1-score (81.10%), precision (73.89%), and AUC 86%. Predictors of incomplete immunization are identified using XGBoost models with help of Shapely additive eXplanation. This study revealed that the number of living children during birth, antenatal care follow-up, maternal age, place of delivery, birth order, preceding birth interval and mothers’ occupation were the top predicting factors of incomplete immunization. Thus, family planning programs should prioritize the number of living children during birth and the preceding birth interval by enhancing maternal education. In conclusion promoting institutional delivery and increasing the number of antenatal care follow-ups by more than fourfold is encouraged.

## Introduction

The national Immunization program is one of the most economically advantageous health treatments, with tested methods for reaching the most vulnerable and difficult-to-reach groups in both developing and developed countries^1–4^. World Health Organization (WHO) launched the Expanded Program Immunization (EPI) in 1974 to ensure universal access to all vaccines for all targeted groups, including children, adolescents, and adults^5^. According to the WHO guidelines, the national EPI now aims to immunize infants between the ages of 0 and 23 months (about 2 years) against eight vaccine-preventable childhood illnesses, such as one dose of measles, three doses of polio, one dose of Bacillus Calmette-Guerin (BCG), and three doses of pentavalent^6^. Hence, a child is fully vaccinated if he/she has received all eight doses of vaccination listed above.

According to the United Nations Children’s Fund (UNICEF) and WHO report in 2019, almost 20 million children were either unvaccinated or had incomplete immunization, making them more susceptible to mortality and morbidity^7^. Of the 20 million children worldwide, who missed vaccination in 2019, over 60% were from 10 countries, many of whom live in countries with weak health systems^7,8^. The global under-five mortality rate declined from 59%, which was 93 deaths per 1000 live births in 1990, to 38% in 2021, due to huge portion of immunization^9^. COVID-19 pandemic and related disruptions have put a burden on health systems, resulting in 25 million children missing out vaccinations in 2021, a number that is 5.9 million higher than in 2019 and the largest amount since 2009^10^.

Although WHO's goal is to make vaccination services accessible to everyone worldwide by 2030, about 13.5 million children did not receive the first dose of a vaccine due lack of access to vaccination services^11^. Ethiopia had over 10.9 million children under the age of one who missed the first dose of measles between 2010 and 2018, which was the highest amount^12^. Through effective vaccination programs, COVID-19 demonstrates the vital role that vaccines play in illness prevention, lifesaving, and promoting a wealthier future^13^.

Even though that Africa has made remarkable progress in immunization services, according to the 2013 immunization data report, vaccine coverage was 75 percent, and Ethiopia has the second largest number of unvaccinated children in the region, next to Nigeria^14^. More children in Africa have lost their immunizations in recent years as the number of births has increased and immunization programs have stagnated^12^. In Ethiopia, the prevalence of complete childhood vaccination status among children aged 12–23 months increased from 24.6 to 39% between 2011 and 2016, respectively^11^. Despite this, according to a recent systematic review and meta-analysis report, one in two children was not vaccinated or four out of ten children had incomplete vaccine in Ethiopia^15^.

Several researches have been conducted to investigate the potential factors associated with incomplete immunization through the application of classical statistical analysis techniques^16–20^ based on prior assumptions that could limit the potential to discover hidden knowledge. In contrast, machine learning algorithms are designed to make the most accurate predictions possible, enabling systems to learn from data rather than making prior assumptions^21^. There are still high rates of incomplete childhood immunization, which require further investigation to prioritize and promote childhood vaccination to ensure the health and well-being of all children in east Africa. Therefore, this research was aimed to predict incomplete immunization among under-five children in East Africa using machine learning algorithms.

## Methods

### Study design and setting

Demographic and Health Survey (DHS) used population based cross-sectional survey study design to collect data and this study employed predictive modeling approach. Secondary data of six east African countries namely Burundi, Ethiopia, Madagascar, Uganda, Rwanda, and Zambia DHS dataset from 2016 to the recent 2021 were considered for this analysis.

### Source and study population

Source population includes all mothers aged 15–49 years who had children under the age of five while all mothers aged 15–49 years who had children under the age of five and started immunization for their children were considered as source population.

### Inclusion criteria

Mothers with children aged 12–35 months who had begun immunization were included in the study.

## Data source, Sample size and sampling procedure

### Data source

Data was obtained from the MEASURE of DHS program^22^. The DHS is a nationally representative survey that collects data on basic health indicators such as mortality, morbidity, family planning service utilization, fertility, maternal and child health services (vaccination). Each country’s survey consisted of different datasets including men, women, children, birth, and household datasets.

### Sample size determination and sampling procedure

A total of 27,806 weighted sample and 27,691 actual sample were considered from six east African countries (Burundi, Ethiopia, Madagascar, Uganda, Rwanda, and Zambia) as shown in Table 1.

**Table 1:** Sample size determination for incomplete immunization in east Africa DHS 2016–2021.

Number	Country	Year	Actual sample size
1.	Burundi	2016–2017	5261
2.	Ethiopia	2016	4083
3.	Madagascar,	2021	4930
4.	Uganda,	2016	6134
5.	Rwanda	2019–20	3276
6.	Zambia	2018	4007
Total			27,691

DHS used two stages of stratified sampling technique to select study participants. In the first stage, Enumeration Areas (EAs) were randomly selected whereas in the second stage households were selected. The survey datasets were accessed through the web page of the International DHS Program after subscription and appropriate letter is acknowledged.

### Study variables

Incomplete immunization in children under the age of five were outcome variable categorized as 1 = Yes (children who had not completed the full dose of vaccination) and 0 = No (those who had received the full dose of vaccination). Baseline explanatory variables were selected from previous studies^14,16,18,23–30^. Thus, sociodemographic factors include mothers age, marital status, mothers’ occupation, mothers’ educational level, husband education, place of residence and sex of household head. socioeconomic factors include wealth index and media exposure while reproductive(obstetrics) history factors include mothers’ history of ANC follow-up, place of delivery, sex of child, number of living children, birth order, child size at birth, PNC visit and preceding birth interval were independent variables.

### Operational definition

Incomplete immunization: “children who started vaccination and missed at least one dose from eight recommended vaccination at any time instance between 1 and 12 months”^31,32^.

Complete immunization: “when children had been vaccinated for all recommended vaccination (one dose of BCG, three doses of polio, three doses of pentavalent, and one dose of measles)”^32^.

### Data management and analysis

Data extraction was carried out using Stata version 17, and then imported to Jupyter Notebook for further analysis. Sample size weighting was used to draw valid inference. Data were thoroughly cleaned, and missing values were imputed to ensure completeness. Outlier detection was performed to identify and remove extreme values that could have skewed the analysis. Python 3 programming language in Jupyter Notebook using imblearn, sklearn^33^, XGBoost^34^ and SHAP^35^ packages were utilized to perform the necessary calculations and analysis.

### Machine learning framework for prediction of incomplete immunization

**Figure 1:**
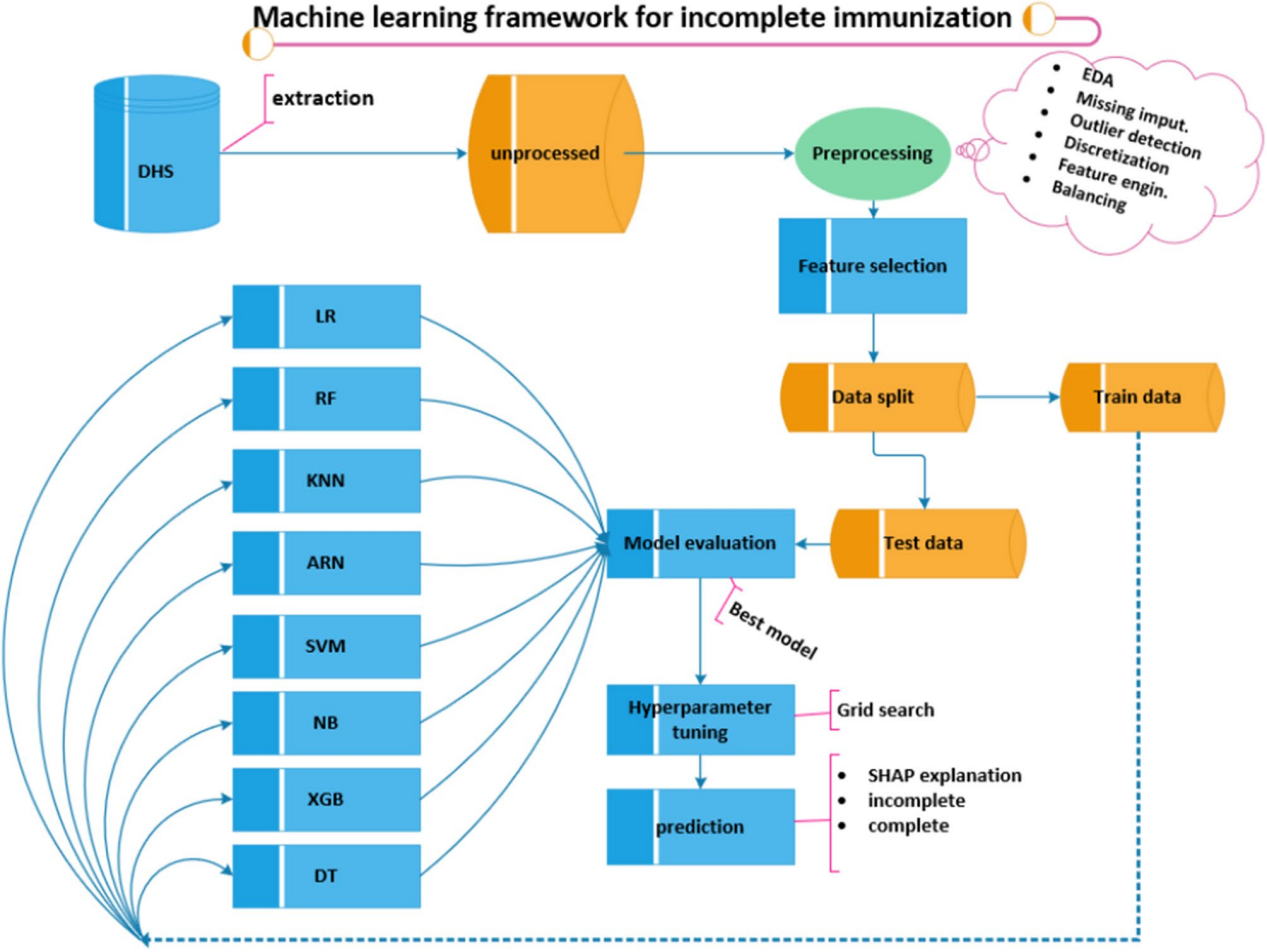
Overview of machine learning framework for prediction of incomplete immunization applied. *LR* logistic regression, *RF* random forest, *KNN* k-nearest neighbor, *ARN* neural network, *SVM* support vector machine, *NB* Naive Bayes, *XGB* eXtreme gradient boosting, *DT* decision tree.

A general framework utilized in earlier research^36^ was created (Fig. 1) based on Yufeng Guo's seven machine learning processes^37^, to predict incomplete vaccination. All machine learning algorithms and techniques were implemented using Python version 3.10.11 programming language in Jupyter Notebook.

### Data collection and preprocessing method

The dataset for this study was extracted from Demographic and Health Survey website and obtained upon a formal request after subscription and registration on their system. A total actual sample of 27,691 under-five children who started vaccination then appropriate data preparation was performed to make data suitable for ML task.

Missing data were managed using various imputation procedures to fill incomplete fields with statistically relevant substitutes. The k-nearest neighbor (KNN) technique has proven to be typically effective for missing value imputation^38^. In this study a simple imputer class of scikit-learn module mode for categorical data and KNN for numerical data were used for imputing missing values in the dataset. Outliers were identified using a boxplot and replaced using the Interquartile Range (IQR) scores for the next step.

Before fitting the ML model, feature engineering was applied. Among various data transformation techniques, we used One Hot Encoder and label Encoder to encode categorical variables into numeric values and min–max normalization technique was used for scaling. Standard balancing strategies including random under-sampling, random over-sampling, and the Synthetic Minority Oversampling Technique (SMOTE) were tested to address the unbalanced categories of the outcome variable. As a result, SMOTE outperformed the other resampling techniques on baseline model.

Following feature engineering dimensionality reduction was applied. High-dimensional data may contain a lot of redundant and useless information, which might seriously reduce how well learning algorithms work^39^. The mutual information and variance threshold from filter method, Recursive Feature Elimination (RFE) from wrapper method and Boruta feature selection method were tested and compared their performance on baseline model for feature selection technique.

Since every ML need training and test dataset, data split was allocated as 80% for training and 20% for testing. The popular k-fold cross validation approach was utilized to ensure the performance of the model because the train-test split function method has disadvantages that it might result in the data being over-fitted or under-fitted on splitted data. In K-fold method, the dataset is split into ‘k’ sub-samples, in which one sample is used for testing and the rest of the k − 1 data set is used for training purpose^33^.

### Model development methods

The dataset used in the analysis falls under the category of binary classification since incomplete immunization is categorized into two mutually exclusive categories. Accordingly, eight classification algorithms (Logistic Regression, Random Forest, K-nearest neighbor (KNN), Artificial Neural Network, Support Vector Machine, Naïve Bayes, eXtreme gradient boosting (XGBoost), and Decision tree) were fitted for this study. These methods were chosen based on prior research that used machine learning techniques for classification tasks using DHS data, with each country's performance taken into account^40–47^.

To verify the algorithm’s performance in terms of classifications, a confusion matrix (also known as an error matrix) and Jaccard score is used. It summarizes the actual and predicted classifications of a dataset and shows the number of correct and incorrect predictions, which are further categorized into true negatives, false negatives, true positives, and false positives. Additionally, the importance and effect of each variable's contribution on the outcome were identified using SHapley Additive exPlanations (SHAP). SHAP is a game theoretic approach to explain the output of any machine learning model that connects optimal credit allocation with local explanations using the classic Shapley values from game theory and their related extensions^35^. Furthermore, receiver operating characteristic curve AUC was used for visualizing summary of performance ML models. Detail of confusion matrix were adapted from^48^ and presented as follows:

**Table:** 

	Predicted positive	Predicted negative
Actual positive	True positive (TP)	False negative (FN)
Actual negative	False positive (FP)	True negative (TN)

Building an efficient ML model is thought to depend heavily on tuning hyper-parameters, especially for tree-based ML models that include a lot of hyper-parameters^49^. It might be challenging to determine what values to use for a particular algorithm's hyperparameters on a specific dataset while the process of searching terminates when predefined criteria are satisfied. For this study, hyper-parameter tunning was done using grid search methods. Finally, supervised ML uses the highest-performing classifier with a defined performance to predict incomplete immunization based on identified independent factors.

It is not over yet because we are still ignorant of the precise feature categories connected to incomplete immunization. For this purpose, rule generation was done using best performed model. Association rules are IF–THEN rules that are particularly significant since they are simple to understand and limit the attributes chosen for the model during rule generation to those that are pertinent^50^.

### Ethics considerations and consent to participate

Since the study was a secondary data analysis, participant consent was not necessary. Permission for data access has been granted from the Demographic and Health Survey (DHS) measure through an online platform by filling all requirements needed to access data from http://www.dhsprogram.com. The IRB-approved procedures for DHS public-use datasets do not allow respondents, households, or sample communities to be identified. There are no names of individuals or household addresses in the data files.

## Results

### Sociodemographic characteristics

**Figure 2:**
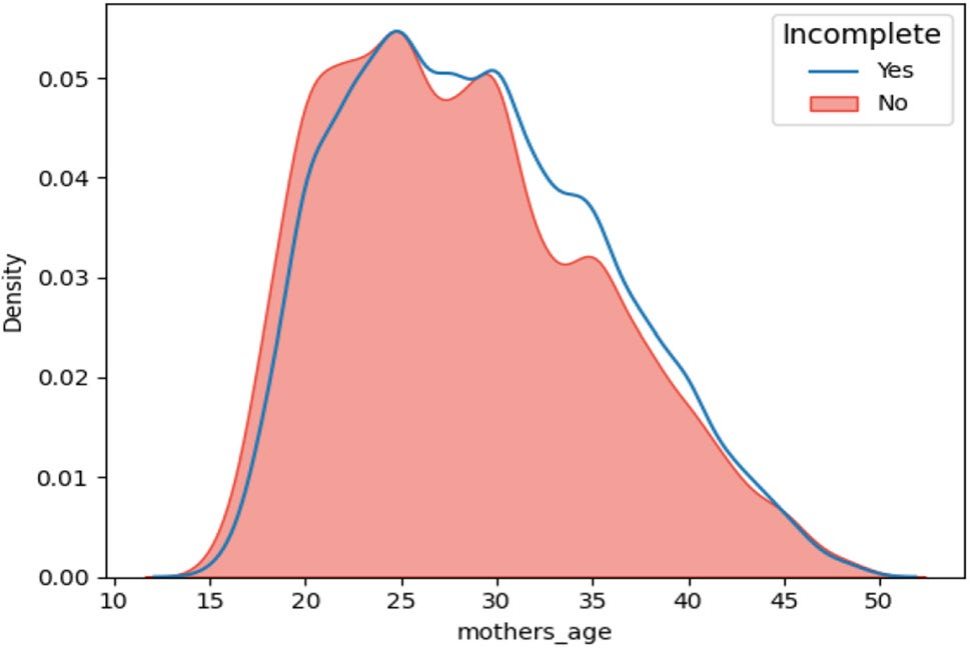
Line plot graph of mothers' age vs immunization status in east Africa DHS 2016–2021.

This study included a total weighted sample of 27,806 children under five. According to the data about 79.54% of participant mothers reside in rural areas. Sex of household head: Males accounted for 79.22% of the household heads. About 20% were female, approximately 85% were married, and only 6.23% and 9% single and widowed or divorced, respectively. Of all mothers in the total country, only 3.62% were professional workers, and most of them (71.78%) were not professional workers. Above half, (55.35%) of husbands took primary education, and still, 21.37% have no regular education. Of all, only 23.28% completed secondary and above level education. Surprisingly, half (49.32% of mothers) took primary education, and 27.25% and 21.37% had no regular education and completed secondary or above by level education, respectively. A summary of sociodemographic characteristics is shown in Table 2 and (Fig. 2) for mothers' ages.

**Table 2:** Sociodemographic characteristics of incomplete immunization among under-five children in east Africa DHS 2016–2021.

Index	Variable	Category	Weighted frequency	%
1	Place of residence	Rural	22,004	79.54
		Urban	5662	20.46
2	Sex of house head	Male	21,916	79.22
		Female	5750	20.78
3	Marital status	Married	23,455	84.77
		Divorced/widowed	2491	9.00
		Single	1720	6.23
4	Mothers’ occupation	Not working	6807	24.60
		Not professional	19,859	71.78
		Professional	1000	3.62
5	Husband education	Primary	15,312	55.35
		Sec &_above	6441	23.28
		No education	5913	21.37
6	Mothers’ education	Primary	13,643	49.32
		No education	7539	27.25
		2ndry &_above	6484	23.43

### Reproductive (obstetrics) history characteristics

The data reveals that the majority of children born to the participants were male, accounting for 51.02% of the total number. Furthermore, most participants did not receive PNC checkup, which is concerning, as it is an important aspect of postnatal care. However, it is reassuring to note that 21.38% of the participants did receive PNC checkup. In terms of place of delivery, most deliveries took place in a health institution, which is a positive indicator of access to healthcare services. However, it is important to note that home deliveries still accounted for a considerable proportion of births, at 32.86%. Finally, the majority of children were of average size at birth accounts 68.93%, while 21.14% were small and 9.93% were large. A summary of reproductive history characteristics is shown in Table 3.

**Table 3:** Reproductive (obstetrics) history characteristics of incomplete immunization in east Africa DHS 2016–2021.

Index	Variable	Category	Weighted frequency	%
1	Sex of child	Male	14,117	51.02
		Female	13,549	48.98
2	PNC checkup	No	21,749	78.62
		Yes	5917	21.38
3	Place of delivery	Health institution	18,575	67.14
		Home	9091	32.86
4	Child size at birth	Average	19,066	68.93
		Small	5850	21.14
		Large	2750	9.93

### Socioeconomic characteristics

According to the data, the wealth index of the participants was distributed as follows: 48.35% were poor, 34.65% were middle class, and 16.99% were rich. It is important to note that many participants were classified as poor, which could have implications for their access to healthcare and other essential services. Regarding media exposure, most participants had access to media, accounting for 51.62% of the total number. However, it is concerning that almost half (48.38%) of the participants did not have access to media, which could limit their access to important health information and education.

### Machine learning analysis of incomplete immunization

**Figure 3:**
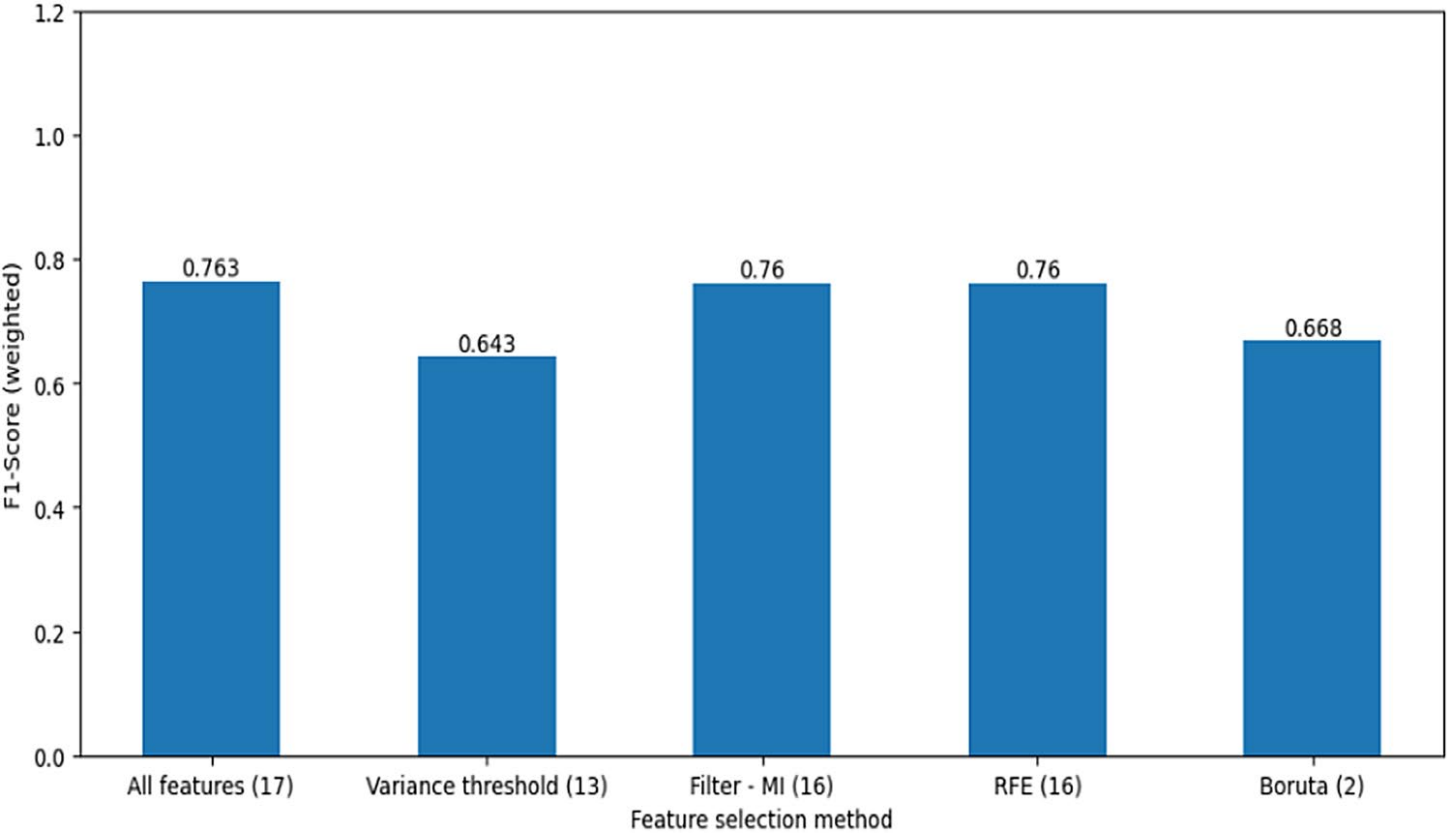
Feature selection methods for incomplete immunization.

This study tried to do feature selection using different techniques to reduce the number of features, as shown in (Fig. 3). Mutual information and variance threshold from the filter method, Recursive Feature Elimination (RFE) from the wrapper method, and Boruta feature selection method were tested and compared their accuracy on baseline model. Despite testing various methods, the highest accuracy was achieved when all features were included in the model development process. This may be attributed to the fact that the original features were already extensive and informative, thus including all of them resulted in the best performance.

### Model development and evaluation

Data were splitted into training and test data after being cleaned and balanced. We allocated 80% of the data for training and 20% for testing. Then we developed eight ML models to predict incomplete immunization. All models were fitted on both unbalanced data and balanced data. Finally, each model’s performance was evaluated and compared in the test set before and after balancing in order to select the best predictive model. Accordingly, high performance was achieved after balancing the target variable shown in Table 4.

**Table 4:** Model performance comparison.

Index	Algorithm	Data status	Accuracy	F1-Score	Jaccard
0	Logistic regression	Balanced	0.617936	0.605258	0.433957
		Unbalanced	0.714565	0.299760	0.176305
1	KNN	Balanced	0.665672	0.691098	0.527998
		Unbalanced	0.678641	0.349332	0.211631
2	Random forest	Balanced	0.783433	0.767696	0.622976
		Unbalanced	0.706745	0.352751	0.214145
3	Neural network	Balanced	0.646718	0.653230	0.485035
		Unbalanced	0.715787	0.379733	0.234365
4	Naive Bayes	Balanced	0.590734	0.575691	0.404190
		Unbalanced	0.690127	0.409133	0.257176
5	XGBoost	Balanced	0.787820	0.762429	0.616069
		Unbalanced	0.711388	0.386494	0.239536
6	Decision Tree	Balanced	0.700948	0.704269	0.543531
		Unbalanced	0.618768	0.400461	0.250360
7	SVM	Balanced	0.634784	0.624842	0.454379
		Unbalanced	0.717498	0.255155	0.146233

After applying the SMOTE balancing technique, the results showed that the random forest and XGBoost models were the best predictive models, having the same performance with an accuracy of 78.34%, f1-scores 76.76%, and Jaccard scores 62.29% for random forest and accuracy 78.78%, f1-score 76.24%, and Jaccard scores 61.16% for XGBoost.

The model that performs best on balanced data was exposed to hyperparameter tuning, which is random forest and XGBoost classifier models. Since both models have roughly the same performance in this study, hyperparameter tuning was applied using the grid search approach to both the random forest classifier and the XGBoost classifier in order to ensure the best model. A Grid Search method with ten-fold cross validation was used to optimize the hyper-parameters of ML models. Since it is not straight forward to select best parameter, ‘criterion’: ‘entropy’, ‘n_estimator’:100, 200, 500, ‘max_depth’: None, 5, 10, ‘max_features’:‘sqrt’, ‘log2’, None were searched and 'max_depth': None, 'max_features': 'log2', 'n_estimators': 500 ‘random state = 0’ were pulled for random forest model. While 'n_estimators': [100, 200, 500], 'max_depth': [3, 5, 10], 'learning_rate': [0.1, 0.01, 0.001], 'subsample': [0.8, 1.0], 'colsample_bytree': [0.8, 1.0], 'random_state': [0, 42] were searched and 'colsample_bytree': 1.0, 'learning_rate': 0.1, 'max_depth': 5, 'n_estimators': 500, 'random_state': 42, 'subsample': 0.8 were pulled for XGBoost model. After implementing this, XGBoost was still able to outperform random forest, therefore it was employed as a prediction model.

### Visualization of feature importance

**Figure 4:**
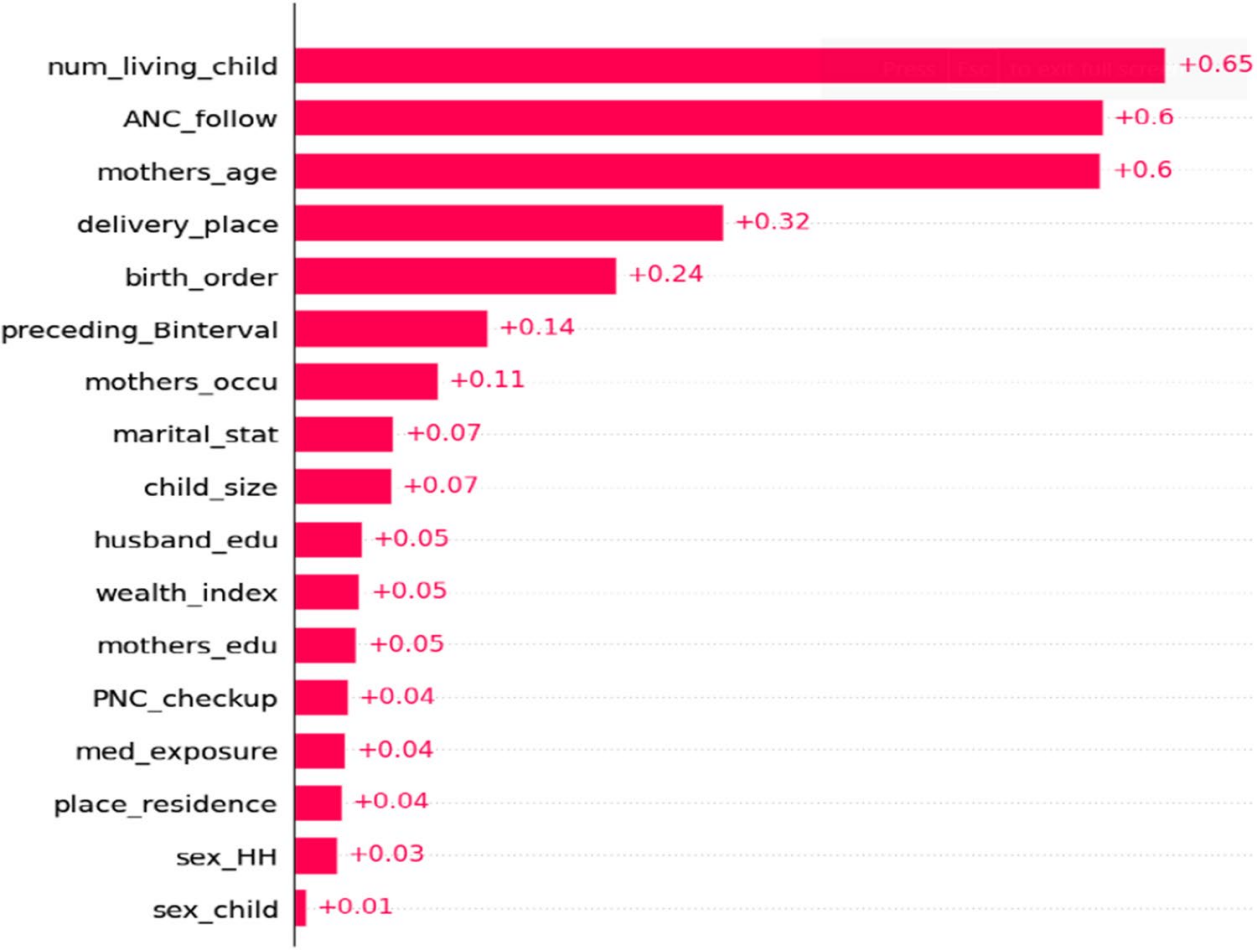
Mean SHAP value of feature importance of incomplete immunization.

While classical analysis is more structured and relies on pre-defined rules and formulas like p-value cut point to select significant features, machine learning algorithms are designed to adapt and learn from data. Although ML models are often considered as black boxes because it is difficult to interpret why an algorithm provides accurate predictions on particular problem^51^; therefore, we introduced the SHAP value in this study. SHAP is a unified framework proposed by Lundberg and Lee^52^ to interpret ML predictions, and it is a new approach to explain various black-box ML models. We leveraged SHAP to explain our predictive model, which includes related predicting factors that lead to incomplete immunization. The importance of predictors is evaluated by the mean SHAP value, as shown on (Fig. 4). Features with a long bar located at the top are highly related to incomplete immunization. Results from feature importance showed, the number of living children during birth, ANC follow-up history, maternal age, place of delivery, birth order, and preceding birth interval were associated with a higher predicted probability of incomplete immunization among under-five children.

**Figure 5:**
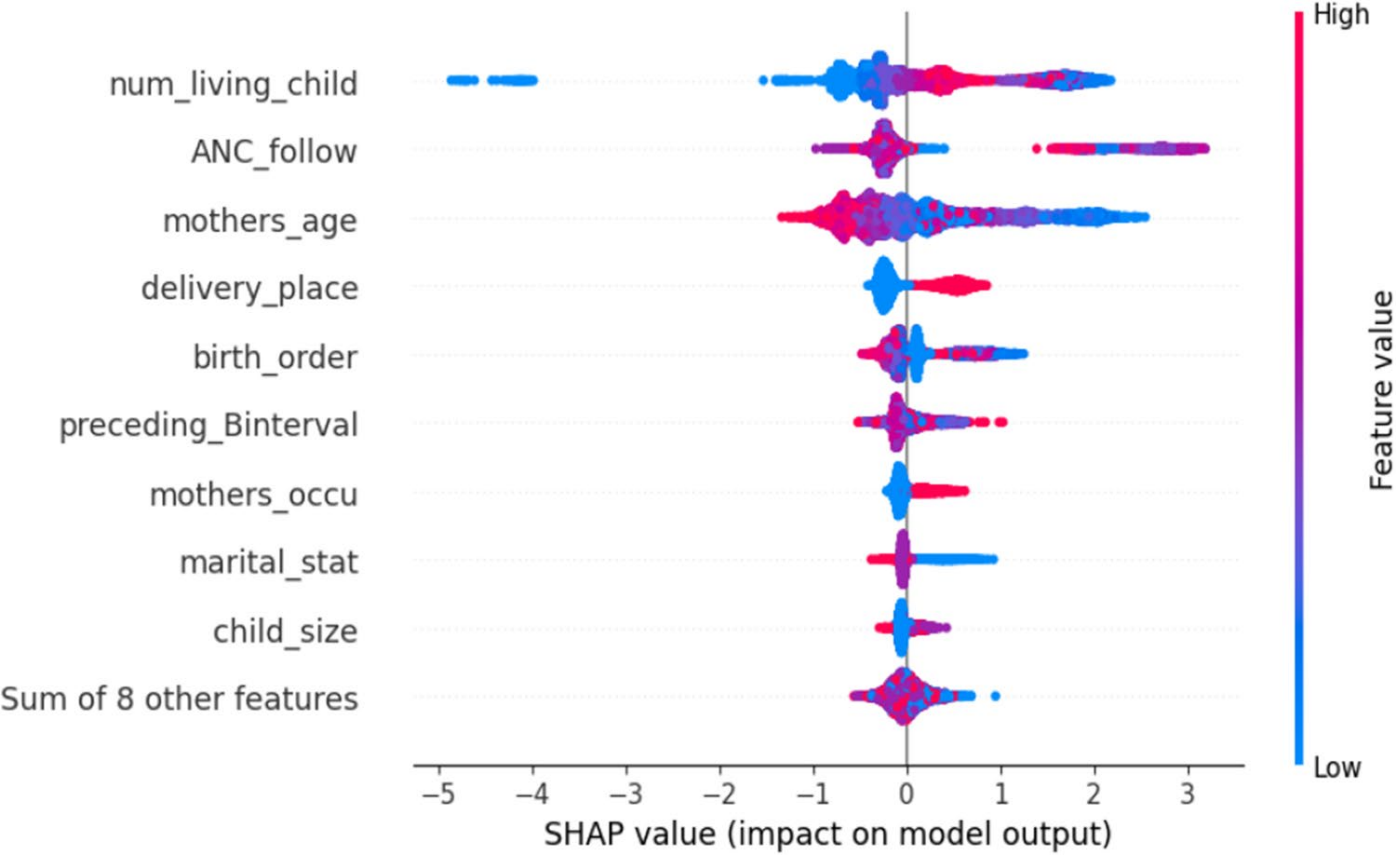
Impact of each variable on prediction of incomplete immunization.

From Fig. 5, the feature ranking (y-axis) indicates the importance of the predictive model. The SHAP value (x-axis) is a unified index that responds to the influence of a certain feature in the model. In each feature important row, the attributions of all variables to the outcome were drawn with dots of distinct colors, where the red dots represent the high-risk value, and the blue dots represent the low-risk value. Features pushing the prediction higher are shown in red, those pushing the prediction lower are in blue. Furthermore, the rest of the other variables had slightly significant effect to low effect on incomplete immunization.

### Predicting incomplete immunization

**Figure 6:**
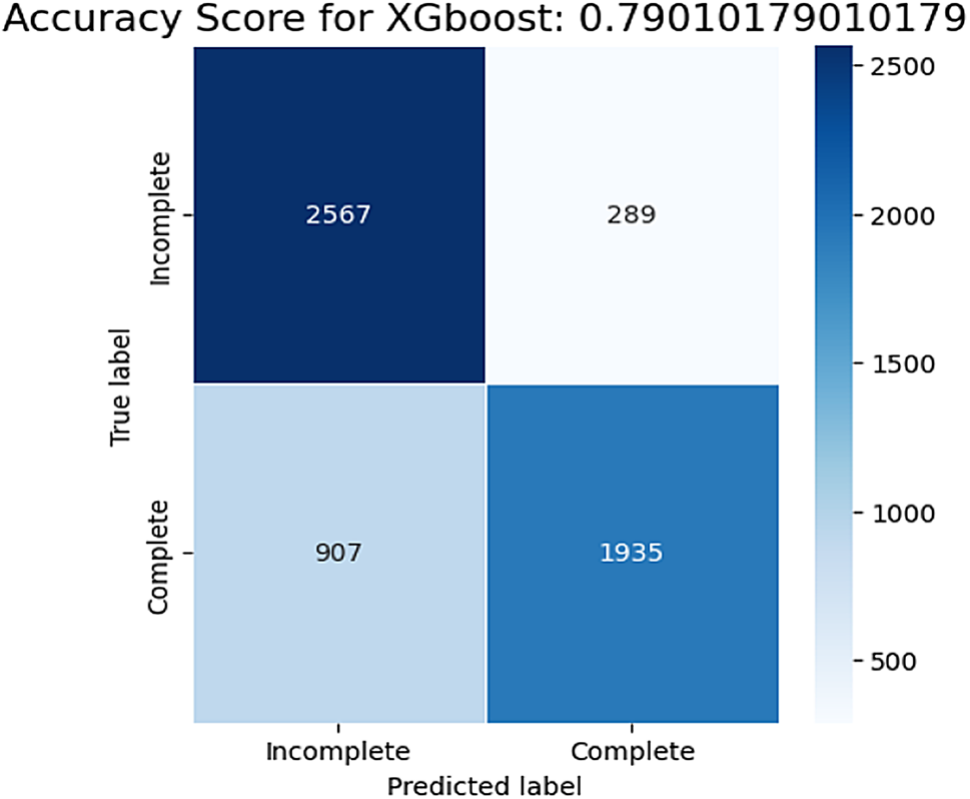
Confusion matrix’s of XGBoost model prediction on test data.

After training, 5698 test samples were used to evaluate the XGBoost model's performance. Out of 2856 incomplete immunization status, the model predicted 2567 of them correctly as incomplete (true positive). And out of 2842 complete, the model predicted 1935 of the as complete (true negative). But the model misclassified 907 true complete immunization status as incomplete (false positive) and 289 true incomplete as complete immunization status (false negative) as shown on (Fig. 6). Overall, the model predicted with an accuracy of 79.01%, recall of 89.88%, F1-score of 81.10%, and 73.89% precision on test data.

$${\mathbf{Accuracy}} = {\text{ TP }} + {\text{ TN }}/ \, \left( {{\text{TP }} + {\text{ FP}} + {\text{FN}} + {\text{TN}}} \right) \, = > { 2567} + {1935 / 2567 } + {289} + {9}0{7} + {1935 } = {\mathbf{79}}.{\mathbf{01}}\%$$

$${\mathbf{Precision}} = {\text{ TP }}/ \, \left( {{\text{TP }} + {\text{ FP}}} \right) = > {2567/ 2567 } + {9}0{7 } = {\mathbf{73}}.{\mathbf{89}}\%$$

$${\mathbf{Recall}} = {\text{ TP }}/ \, \left( {{\text{TP }} + {\text{ FN}}} \right) = > {2567/ 2567 } + {289 } = {\mathbf{89}}.{\mathbf{88}}\%$$

$${\mathbf{F1}}\,{\mathbf{score}} = \, \left( {{2 } \times {\text{ Precision }} \times {\text{ Recall}}} \right) \, / \, \left( {{\text{Precision }} + {\text{ Recall}}} \right) \, \left( {{2} \times 0.{8988} \times 0.{7389}} \right) \, / \, 0.{8988 } + \, 0.{7389 } = {\mathbf{81}}.{\mathbf{10}}\%$$

**Figure 7:**
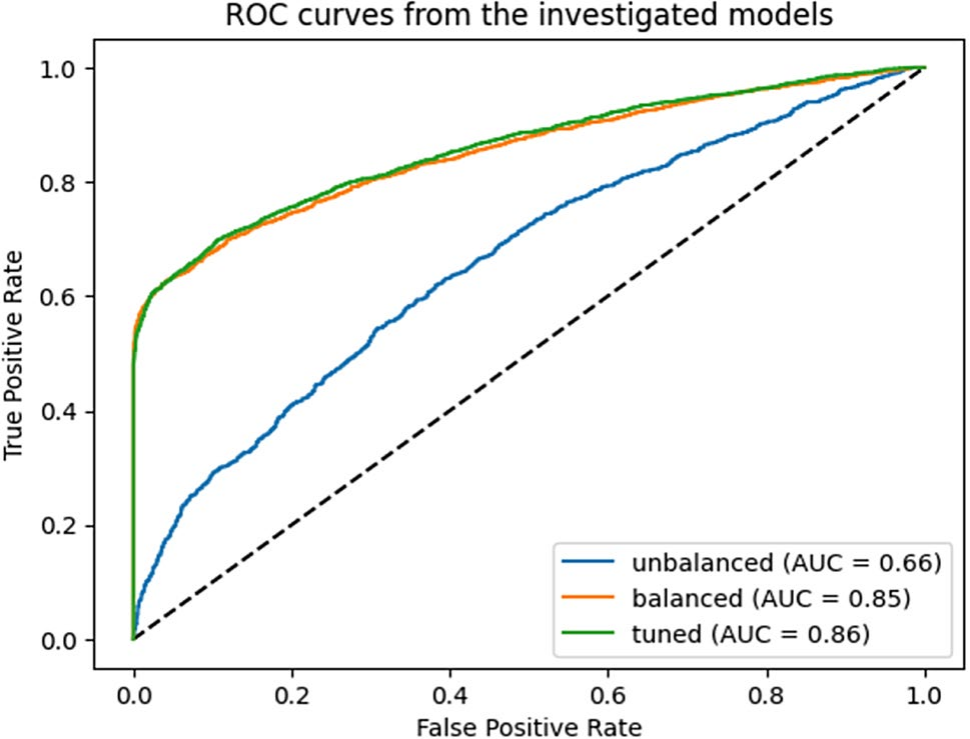
Comparison of XGBoost model prediction on test data.

Area under the receiver operating characteristic curve (AUC) (Fig. 7) was used to summarize model performance overall thresholds and thus misclassification error weightings. XGBoost model produced an area under the curve of 66% on unbalanced data, whereas after balancing and hyperparameter tuning, the prediction on test data produced an area under the curve of 86% which indicates a good predicting model. Below the figure green line shows the model after balancing and tuning while the orange line shows AUC on unbalanced data.

### Association rule mining

Association rule mining is a technique used to discover interesting relationships between variables in large datasets^53^. For this study association rule mining was done using Apriori algorithm to identify the precise category that is linked with incomplete immunization. Before applying association rule mining data discretization was performed for the variables that were not categorical at all. Thus, mothers’ age was categorized as (15–24, 25–34, 35–49). Number of living children categorized as (1–3, 4–6, and > 6). Preceding birth interval categorized as (< 25, 25–48, > 48). ANC follow up categorized as (no visit, 1–4 and > 4). Birth order is categorized as (1st, 2nd and 3rd and above 3rd). Apriori algorithm produces 13 rules connected to target category 1 that replace incomplete immunization status with more than 70% confidence level, but only four rules with more than 85% confidence level were generated.

Rule1 ***IF*** ('mothers_age_15-24’, ‘delivery_place_home', ‘ANC_follow_1-4' ***THEN*** target_1 confidence 89.9% lift 1.696.

Rule2 ***IF*** ('mothers_age_15-24', ‘ANC_follow_1-4', 'mothers_edu_Nedu’, ‘delivery_place_home') ***THEN*** target_1 confidence 87.4% lift 1.695.

Rule3 ***IF*** 'mothers_age_15-24', 'delivery_place_home', 'mothers_edu_Nedu') ***THEN*** target_1 confidence 85.58% lift 1.678.

Rule4 ***IF*** ‘ANC_follow_1-4','delivery_place_home', 'mothers_edu_Nedu') ***THEN*** target_1 confidence 85.5 lift 1.

## Discussion

Recently COVID-19 pandemic and related disruptions have put a burden on the health systems. This results in 25 million children missing out vaccinations in 2021, a number that is 5.9 million higher than in 2019 and the largest amount since 2009^10^. This study was conducted to predict top risk factors of incomplete immunization among children under five. Eight supervised machine learning algorithms were trained on both balanced and imbalanced data for prediction purposes. The performance of those eight ML models was compared by their classification accuracy, f1-score and Jaccard score. SMOTE's data balancing approach outperformed models developed using unbalanced data in terms of accuracy, f1 score, Jaccard score and Area under curve score. In this study XGBoost and random forest performed best same result on balanced data. But after applying hyperparameter XGBoost model improved performance over the random forest with an accuracy of 79.01%, recall of 89.88%, F1-score of 81.10%, precision 73.89%, and AUC 86% while random forest was chosen on study conducted in Sindh province, Pakistan^54^ on predicting elevated risk of defaulting from immunization. This may be due to the fact that they did not test XGBoost model on their research. Final prediction was made on test data after optimizing hyperparameters of XGBoost classifier in turn improved AUC. The model predicted 2567 true positive (true case of incomplete immunization) 1935 true negative (true complete immunization) and misclassified 289 as complete and 907 as incomplete.

Accordingly, top features were identified by SHAP mean value based on their importance in predicting incomplete immunization after model is tuned on XGBoost. In addition, the contribution of each feature to the prediction for incomplete immunization and model accuracy, were identified using SHAP impact (on model output). Those with red dots have high predictive probability or pushing the prediction higher, in contrast feature located at the bottom of tree explainer with blue color were low predictive probability or pushing the prediction lower. This research found number of living children during birth, ANC follow-up history, maternal age, place of delivery, birth order, preceding birth interval were the top associated with a higher predicted probability or pushing factor to incomplete immunization among under five children in east Africa. This result is supported by previous studies done in east Africa using multilevel analysis have shown that factors such as birth order, ANC follow up, place of delivery, preceding birth interval, maternal age have profound influence on mother’s health-seeking behavior and child immunization status^16^.

Another aim of this research was to identify specific categories that are associated with incomplete immunization. Association rule mining was employed to identify which category is more associated with incomplete immunization among children under five in east Africa. The analysis revealed that children whose mothers had no education, were delivered at home instead of a health institution and ANC follow (1–4 times) were highly associated with predictive probability to have incomplete immunization. Additionally, the study found that younger mothers (15–24) were also associated with incomplete immunization.

In our research findings, younger mothers (15–24) were associated with incomplete immunization among children under five in east Africa. This may be due the fact that older mothers had childcare experience which the young mothers are yet to acquire. Additionally, older women may be more willing to continue immunizing their children since they may have previously had children who received vaccinations and had no negative side effects. Similarly, a study conducted in Nigeria^18^ Ethiopia^14^ and Kenya^55^ agreed that Children of young women (15–24 years) are more likely to be incompletely immunized when compared with children of older women. Possible explanation could be this study attributed to large samples and included more areas beyond Ethiopia.

Results from rule generation revealed that children whose mothers had no education were associated with incomplete immunization. A similar association between maternal education and child immunization has been reported in several other studies, including Togo^56^ Nigeria^18^ Athens Greece^57^ Hadiya zone, Ethiopia^58^ systematic review across the globe^59^. Indeed, a woman's education has a demonstrable impact on her ability to acquire information about the usage of health services in general and vaccination services in particular, as well as her level of living. According to research, education has a significant impact on mothers' health-seeking habits, including child vaccination^18^. Education also makes it simpler for women to communicate with medical experts, leading to a better understanding of and ability to absorb knowledge about actions that enhance children's welfare. In contrast study conducted in rural of Mozambique showed Mothers' educational levels had no influence on the child's vaccination status^60^. This may be due study conducted only on rural area since residence variation have impact on mothers’ education related factor like media exposure in addition small sample size is not representative which led to bias.

Home delivery was associated with incomplete child immunization in East Africa. This finding is in line with the studies conducted in Nepal^61^ India^62^ Tigray northern Ethiopia^63^ Madagascar^64^ Kenya^55^. Similarly home delivery was reported to be a risk factor in case–control studies^65^ and systematic review^66^ conducted in Ethiopia. The explanation could be women who give birth at a home are less likely to be aware of their own and their children’s health status than institutional delivery. According to a systematic review and meta-analysis conducted in Ethiopia, women who gave birth at home were 3 times more likely to have incompletely immunized children than women who delivered at health facilities^67^.

Our study also shows that the utilization of health services such as ANC can be an important factor for the incompleteness of children’s vaccination status. This is consistent with the study conducted in Tanzania^68^ Senegal^69^ and a previous study in East Africa by multilevel analysis^16^. This might be explained by the fact that mothers obtaining sufficient helpful information about kid immunizations at ANC visits, giving them confidence in their children's preventive health. This result, also supported by systematic review and meta-analysis from Ethiopia, revealed that ANC follow-up services were found to be significantly associated with incomplete vaccination^67^. Indeed, ANC follow-up is important for child immunization as it allows healthcare providers to monitor the mother's health during pregnancy, ensuring that the child receives the necessary vaccinations.

### Strength and limitation of the study

This study has the following limitations. Frist recall and social desirability biases. Although the DHS program is typically regarded as one of the most trustworthy sources of quantitative data, particularly maternal and child health, it may be that the responses were affected by recall and social desirability biases. While acknowledging these and other limitations inherent in national demographic surveys of this kind, the surveys still offer the greatest population-based data currently available, encompassing all the nation's provinces and regions and guaranteeing external validity or generalizability.

Nevertheless, this research has several strengths, one of which is the utilization of machine learning techniques that learn from data rather than relying on prior assumptions as in classical analysis methods. Furthermore, this study provides an invaluable contribution to immunization status literature in context of machine learning.

## Conclusion

This study was conducted with aim of predicting and identifying predicting factors of incomplete immunization in east Africa. Using SHAP mean values and SHAP plots, we proved that the ML method can illustrate the influence of key features and establish a high-accuracy incomplete immunization prediction model. The illustration of cumulative domain-specific feature importance and visualized interpretation of feature importance can allow policy makers and immunization program manager on respective study area to intuitively understand the decision-making process for incomplete immunization among under-five children. Prior to this, number of living children during birth, ANC follow-up history, maternal age, place of delivery, birth order, preceding birth interval must all be taken into consideration while implementing health policies intended to reduce the incomplete immunization. Family planning programs should focus on the number of living children during births and preceding birth interval, by enhancing mothers’ education for respective country. We highly recommend promoting institutional delivery and increasing the number of ANC follow-ups by more than four times. It is essential that all stakeholders like Eastern Africa regional coordination center (RCC) take appropriate measurements to ensure that the immunization process is accessible to all children in the country.

## Data Availability

The dataset used and analyzed in this study is available from the DHS program official database (http://dhsprogram.com) upon formal request. Approval for dataset access is typically confirmed via email.

## Abbreviations

AUC: Area under the curve
COVID-19: Coronavirus disease of 2019
DHS: Demographic and health survey
EA: Enumeration area
EPI: Expanded immunization program
FN: False negative
FP: False positive
IA: Immunization agenda
KNN: K-nearest neighbour
ML: Machine learning
ROC: Receiver operating characteristic
SHAP: SHapley Additive exPlanations
SMOTE: Synthetic minority oversampling technique
TN: True negative
TP: True positive
WHO: World health organization (WHO)
XGBoost: EXtreme gradient boosting

## References

[1] Miller, M. A. & Hinman, A. R. In *Vaccines*, 6th edn (eds Plotkin, S. A., Orenstein, W. A., & Offit, P. A.) 1413–1426 (W.B. Saunders, 2013).

[2] Ozawa S (2016). Return on investment from childhood immunization in low- and middle-income countries, 2011–20. Health Aff. (Project Hope).

[3] Bloom, D. E. In *Hot Topics in Infection and Immunity in Children VII* (eds Curtis, N., Finn, A., & Pollard, A. J.) 1–8 (Springer, 2011).

[4] Sim SY, Watts E, Constenla D, Brenzel L, Patenaude BN (2020). Return on investment from immunization against 10 pathogens in 94 low- and middle-income countries, 2011–30. Health Aff. (Project Hope).

[5] Machingaidze S, Wiysonge CS, Hussey GD (2013). Strengthening the expanded programme on immunization in Africa: Looking beyond 2015. PLoS Med..

[6] Masud, T. & Navaratne, K. V. The expanded program on immunization in Pakistan: Recommendations for improving performance. (2012).

[7] WHO/UNICEF. Progress and challenges with achieving universal immunization coverage. (2020).

[8] WHO and UNICEF: Progress and Challenges with Achieving Universal Immunization Coverage. (WHO/UNICEF Estimates of National Immunization Coverage, J., 2019).

[9] UNICEF. *Under Five Mortality*. https://data.unicef.org/topic/child-survival/under-five-mortality/ (2023).

[10] WHO/UNICEF. *Estimates of National Immunization Coverage*. http://www.who.int/news-room/fact-sheets/detail/immunization-coverage (2021).

[11] Debie A, Lakew AM, Tamirat KS, Amare G, Tesema GA (2020). Complete vaccination service utilization inequalities among children aged 12–23 months in Ethiopia: A multivariate decomposition analyses. Int. J. Equity Health.

[12] UNICEF. (2020).

[13] Faisal, S. *et al.* Modeling the factors associated with incomplete immunization among children. *Math. Probl. Eng.* **2022** (2022).

[14] Negussie A, Kassahun W, Assegid S, Hagan AK (2016). Factors associated with incomplete childhood immunization in Arbegona district, southern Ethiopia: A case-control study. BMC Public Health.

[15] Nour TY (2020). Predictors of immunization coverage among 12–23 month old children in Ethiopia: Systematic review and meta-analysis. BMC Public Health.

[16] Tesema GA, Tessema ZT, Tamirat KS, Teshale AB (2020). Complete basic childhood vaccination and associated factors among children aged 12–23 months in East Africa: A multilevel analysis of recent demographic and health surveys. BMC Public Health.

[17] Skull SA, Ngeow JYY, Hogg G, Biggs B-A (2008). Incomplete immunity and missed vaccination opportunities in East African immigrants settling in Australia. J. Immigr. Minor. Health.

[18] Adedokun ST, Uthman OA, Adekanmbi VT, Wiysonge CS (2017). Incomplete childhood immunization in Nigeria: A multilevel analysis of individual and contextual factors. BMC Public Health.

[19] Russo G (2015). Vaccine coverage and determinants of incomplete vaccination in children aged 12–23 months in Dschang, West Region, Cameroon: A cross-sectional survey during a polio outbreak. BMC Public Health.

[20] Mohamud Hayir TM, Magan MA, Mohamed LM, Mohamud MA, Muse AA (2020). Barriers for full immunization coverage among under 5 years children in Mogadishu, Somalia. J. Fam. Med. Prim. Care.

[21] Kebede Kassaw AA (2023). Spatial distribution and machine learning prediction of sexually transmitted infections and associated factors among sexually active men and women in Ethiopia, evidence from EDHS 2016. BMC Infect. Dis..

[22] DHS. *Data Collection*. https://www.dhsprogram.com/Data/.

[23] Etana B, Deressa W (2012). Factors associated with complete immunization coverage in children aged 12–23 months in Ambo Woreda, Central Ethiopia. BMC Public Health.

[24] Kassahun MB, Biks GA, Teferra AS (2015). Level of immunization coverage and associated factors among children aged 12–23 months in Lay Armachiho District, North Gondar Zone, Northwest Ethiopia: A community based cross sectional study. BMC. Res. Notes.

[25] Sheikh N (2018). Coverage, timelines, and determinants of incomplete immunization in Bangladesh. Trop. Med. Infect. Dis..

[26] Bugvi AS (2014). Factors associated with non-utilization of child immunization in Pakistan: Evidence from the Demographic and Health Survey 2006–07. BMC Public Health.

[27] Tadesse H, Deribew A, Woldie M (2009). Predictors of defaulting from completion of child immunization in south Ethiopia, May 2008—A case control study. BMC Public Health.

[28] Jani JV, De Schacht C, Jani IV, Bjune G (2008). Risk factors for incomplete vaccination and missed opportunity for immunization in rural Mozambique. BMC Public Health.

[29] De P, Bhattacharya BN (2002). Determinants of child immunization in fourless-developed states of North India. J. Child Health Care.

[30] Rahman M, Obaida-Nasrin S (2010). Factors affecting acceptance of complete immunization coverage of children under five years in rural Bangladesh. Salud pública de méxico.

[31] Atnafu A (2020). Prevalence and determinants of incomplete or not at all vaccination among children aged 12–36 months in Dabat and Gondar districts, northwest of Ethiopia: Findings from the primary health care project. BMJ Open.

[32] Melaku MS, Nigatu AM, Mewosha WZ (2020). Spatial distribution of incomplete immunization among under-five children in Ethiopia: Evidence from 2005, 2011, and 2016 Ethiopian Demographic and health survey data. BMC Public Health.

[33] Pedregosa F (2011). Scikit-learn: Machine learning in Python. J. Mach. Learn. Res..

[34] Chen, T. & Guestrin, C. *XGBoost: A Scalable Tree Boosting System*. (2016).

[35] Lundberg SM (2020). From local explanations to global understanding with explainable AI for trees. Nat. Mach. Intell..

[36] Rawat S, Rawat A, Kumar D, Sabitha AS (2021). Application of machine learning and data visualization techniques for decision support in the insurance sector. Int. J. Inf. Manag. Data Insights.

[37] Guo, Y. The 7 steps of machine learning (2017). *towardsdatascience.com* (2017).

[38] Brownlee, J. *Data Preparation for Machine Learning: Data Cleaning, Feature Selection, and Data Transforms in Python* (Machine Learning Mastery, 2020).

[39] Yu, L. & Liu, H. In *Proceedings of the 20th International Conference on Machine Learning (ICML-03).* 856–863.

[40] Bekele WT (2022). Machine learning algorithms for predicting low birth weight in Ethiopia. BMC Med. Inform. Decis. Mak..

[41] Bitew, F. H., Sparks, C. S. & Nyarko, S. H. Machine learning algorithms for predicting undernutrition among under-five children in Ethiopia. *Public Health Nutr.* 1–12 (2021). 10.1017/S1368980021004262 PMC8883776 34620263

[42] Chilyabanyama ON (2022). Performance of machine learning classifiers in classifying stunting among under-five children in Zambia. Children (Basel, Switzerland)..

[43] Emmanuel, M. *Application of Machine Learning Methods in Analysis of Infant Mortality in Rwanda: Analysis of Rwanda Demographic Health Survey 2014–15 Dataset* (University of Rwanda, 2021). 10.1186/s12884-022-04699-8 PMC9066935 35509018

[44] Fenta HM, Zewotir T, Muluneh EK (2021). A machine learning classifier approach for identifying the determinants of under-five child undernutrition in Ethiopian administrative zones. BMC Med. Inform. Decis. Mak..

[45] Kananura RM (2022). Machine learning predictive modelling for identification of predictors of acute respiratory infection and diarrhoea in Uganda’s rural and urban settings. PLoS Glob. Public Health.

[46] Saroj RK, Yadav PK, Singh R, Chilyabanyama ON (2022). Machine learning algorithms for understanding the determinants of under-five mortality. BioData Min..

[47] Tesfaye B, Atique S, Azim T, Kebede MM (2019). Predicting skilled delivery service use in Ethiopia: Dual application of logistic regression and machine learning algorithms. BMC Med. Inform. Decis. Mak..

[48] Bekkar M, Djemaa HK, Alitouche TA (2013). Evaluation measures for models assessment over imbalanced data sets. J. Inf. Eng. Appl..

[49] Yang L, Shami A (2020). On hyperparameter optimization of machine learning algorithms: Theory and practice. Neurocomputing.

[50] Kebede SD (2023). Prediction of contraceptive discontinuation among reproductive-age women in Ethiopia using Ethiopian Demographic and Health Survey 2016 Dataset: A machine learning approach. BMC Med. Inform. Decis. Mak..

[51] Wang K (2021). Interpretable prediction of 3-year all-cause mortality in patients with heart failure caused by coronary heart disease based on machine learning and SHAP. Comput. Biol. Med..

[52] Lundberg, S. M. & Lee, S.-I. A unified approach to interpreting model predictions. *Adv. Neural Inf. Process. Syst.* **30** (2017).

[53] Li Q, Zhang Y, Kang H, Xin Y, Shi C (2017). Mining association rules between stroke risk factors based on the Apriori algorithm. Technol. Health Care..

[54] Chandir S (2018). Using predictive analytics to identify children at high risk of defaulting from a routine immunization program: Feasibility study. JMIR Public Health Surveill..

[55] Mutua MK, Kimani-Murage E, Ettarh RR (2011). Childhood vaccination in informal urban settlements in Nairobi, Kenya: Who gets vaccinated?. BMC Public Health.

[56] Landoh DE (2016). Predictors of incomplete immunization coverage among one to five years old children in Togo. BMC Public Health.

[57] Pavlopoulou ID, Michail KA, Samoli E, Tsiftis G, Tsoumakas K (2013). Immunization coverage and predictive factors for complete and age-appropriate vaccination among preschoolers in Athens, Greece: A cross- sectional study. BMC Public Health.

[58] Zewdie A, Letebo M, Mekonnen T (2016). Reasons for defaulting from childhood immunization program: A qualitative study from Hadiya zone, Southern Ethiopia. BMC Public Health.

[59] Tauil MDC, Sato APS, Waldman EA (2016). Factors associated with incomplete or delayed vaccination across countries: A systematic review. Vaccine.

[60] Shrestha S, Shrestha M, Wagle RR, Bhandari G (2016). Predictors of incompletion of immunization among children residing in the slums of Kathmandu valley, Nepal: A case-control study. BMC Public Health.

[61] Chhabra P, Nair P, Gupta A, Sandhir M, Kannan AT (2007). Immunization in urbanized villages of Delhi. Indian J. Pediatr..

[62] Aregawi HG, Gebrehiwot TG, Abebe YG, Meles KG, Wuneh AD (2017). Determinants of defaulting from completion of child immunization in Laelay Adiabo District, Tigray Region, Northern Ethiopia: A case-control study. PLoS One.

[63] Verrier F (2023). Vaccination coverage and risk factors associated with incomplete vaccination among children in Cambodia, Madagascar, and Senegal. Open Forum Infect. Dis..

[64] Tesfaye, F., Tamiso, A., Birhan, Y. & Tadele, T. Predictors of immunization defaulting among children age 12–23 months in Hawassa Zuria District of southern Ethiopia: Community based unmatched case control study. *Int. J. Public Health* **3** (2014).

[65] Atnafu Gebeyehu, N. *et al.* Incomplete immunization and its determinants among children in Africa: Systematic review and meta-analysis. *Hum. Vaccines Immunother*. 10.1080/21645515.2023.2202125 (2023). 10.1080/21645515.2023.2202125 PMC10294773 37144686

[66] Desalew, A., Semahegn, A., Birhanu, S. & Tesfaye, G. Incomplete vaccination and its predictors among children in Ethiopia: A systematic review and meta-analysis. *Glob. Pediatr. Health* **7**, 2333794x20968681. 10.1177/2333794x20968681 (2020). 10.1177/2333794X20968681 PMC7675896 33241080

[67] Mrisho M (2009). The use of antenatal and postnatal care: perspectives and experiences of women and health care providers in rural southern Tanzania. BMC Pregnancy Childbirth.

[68] Mbengue MAS (2017). Determinants of complete immunization among senegalese children aged 12–23 months: Evidence from the demographic and health survey. BMC Public Health.

[69] Sarker AR, Akram R, Ali N, Chowdhury ZI, Sultana M (2019). Coverage and determinants of full immunization: Vaccination coverage among senegalese children. Medicina (Kaunas, Lithuania)..

